# Minimally Invasive Resection of Giant Neurocysticercal Cyst

**DOI:** 10.4269/ajtmh.22-0100

**Published:** 2022-05-23

**Authors:** Paul Albert Trubin, Sacit Bulent Omay, Albert I. Ko

**Affiliations:** ^1^ Department of Internal Medicine, Section of Infectious Diseases, Yale University School of Medicine, New Haven, Connecticut;; ^2^ Department of Neurosurgery, Yale University School of Medicine, New Haven, Connecticut;; ^3^ Department of Epidemiology of Microbial Diseases, Yale School of Public Health, New Haven, Connecticut

A 55-year-old man from Mexico with history of diabetes mellitus presented to the emergency department with report of worsening dizziness over the preceding several weeks along with progressive gait instability. Despite these symptoms, he remained alert and oriented, and continued working in food preparation.

On admission, computed tomography of the head revealed a large, cystic mass situated within the right frontal lobe associated with surrounding edema. Significant right-to-left midline shift, subfalcine herniation, and uncal crowding were noted. Magnetic resonance imaging (MRI) revealed a bilobed, cystic lesion 8.8 cm in largest dimension with surrounding edema and mass effect (Figure 1A and B). The differential diagnosis included malignant or infectious processes. The patient had emigrated from rural Mexico to Connecticut 20 years before hospitalization. He reported regular exposure in early childhood to farm animals. He consumed pork infrequently.

**Figure 1.**
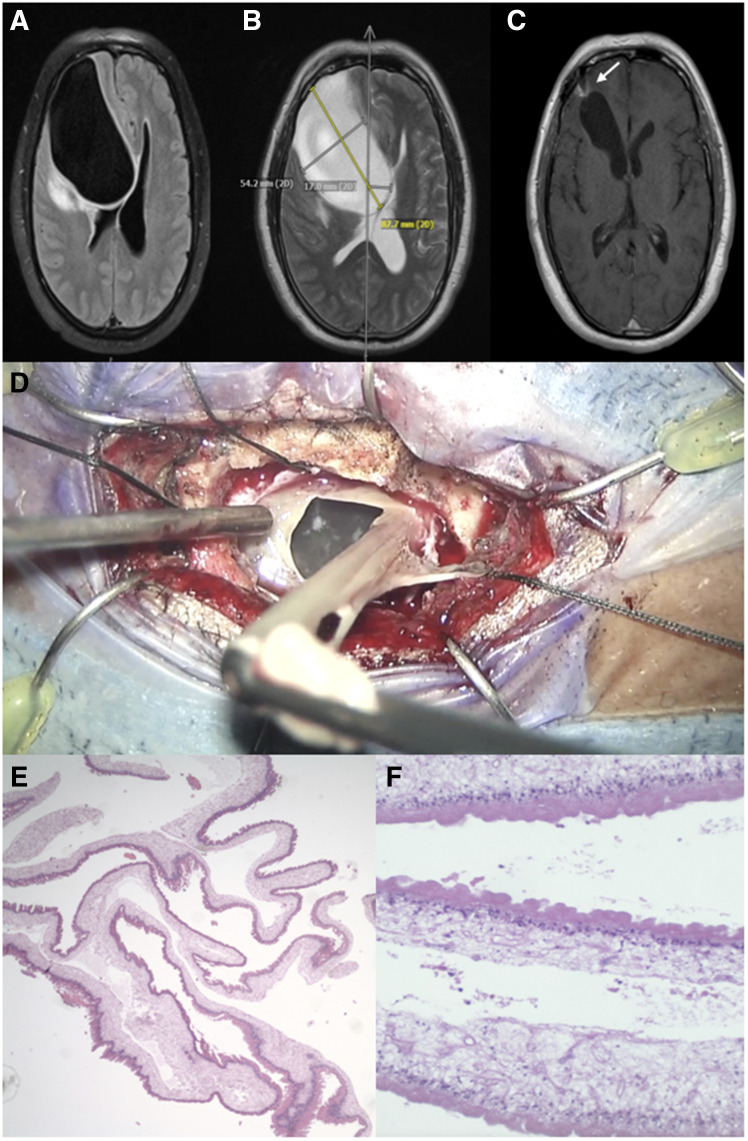
Giant racemose neurocysticercosis. (**A**) T1-weighted axial magnetic resonance imaging displaying 8.8-cm superior lobe of a bilobed frontal cystic lesion. (**B**) T2-weighted axial magnetic resonance imaging displaying 8.8-cm superior lobe of a bilobed frontal cystic lesion. (**C**) T1-weighted axial magnetic resonance imaging displaying collapse of frontal lesion (arrow) and hydrocephalus *ex vacuo* 9 months postoperatively. (**D**) Intraoperative still of cyst excision via minimally invasive supraorbital craniotomy and endoscopic exploration of remnant cavity. (**E**) Surgical pathology specimen showing cestode cyst wall, hematoxylin and eosin staining (40× magnification). (**F**) Surgical pathology specimen showing cestode cyst wall, hematoxylin and eosin staining, with outer cuticular, cellular, and reticular layers (200× magnification). This figure appears in color at www.ajtmh.org.

Given the differential diagnosis, the patient underwent minimally invasive supraorbital craniotomy and resection of the lesion to decompress the mass effect and to secure a pathology-based diagnosis (Figure 1D, Supplemental Video). A spinal needle was used to access cyst contents through a brown-colored pseudocapsule after retraction of dura. A whitish-colored underlying capsular structure herniated from this puncture site. To accommodate the herniation, the outer pseudocapsule was opened. The whitish-colored cystic structure was removed. An additional component of the cystic structure near the frontal base was accessed, yielding clear yellow fluid, and explored endoscopically. Frozen intraoperative histopathology was suggestive preliminarily of a cestode cyst.

Microscopy of the cyst wall was consistent with neurocysticercosis (Figure 1E and F); a scolex was not identified. Racemose neurocysticercosis was diagnosed on the basis of clinical, imaging, and histopathologic data. *Taenia solium* serum antibody returned positive. The patient was treated with praziquantel, albendazole, and steroid therapy.^1^^,^^2^ Three months of antihelminthic therapy and a tapered steroid regimen were completed. Follow-up MRI studies indicated collapse of the lesion (Figure 1C), and presenting symptoms resolved. 1.5 years after hospitalization, generalized seizures developed, thought to be secondary to gliosis. MRI ruled out cyst recurrence, and antiepileptic therapy was initiated.

The giant, racemose cystic lesion is a rarer manifestation of neurocysticercosis. Treatment options include both medical and surgical interventions.^1^^,^^3^^,^^4^ Factors influencing treatment decisions include the presence and degree of symptoms, and the number, viability, and anatomical location of cystic lesions; treatment decisions should be tailored to individual cases.^2^ There has been increasing experience using minimally invasive surgical approaches for extirpation of large cysts, particularly involving the ventricular and subarachnoid compartments, which may provide improved postoperative outcomes when surgery is required.^5^

## Supplemental Material


Supplemental materials
